# Vascular adhesion protein-1 expression is reduced in the intestines of infants with necrotizing enterocolitis: an observational research study

**DOI:** 10.1186/s12887-022-03681-9

**Published:** 2022-11-05

**Authors:** Björn Andersson, Laszlo Markasz, Hamid Mobini-Far, Helene Engstrand Lilja

**Affiliations:** 1 Department of Women’s and Children’s Health, Uppsala University, Dag Hammarskjölds Väg 14B, 752 37 Uppsala, Sweden; 2 Department of Pediatric Surgery, University Children’s Hospital, Uppsala, Sweden; 3 Neonatology Division, University Children’s Hospital, Uppsala, Sweden; 4 Department of Pathology, Uppsala University Hospital, Uppsala, Sweden

**Keywords:** Infant, Necrotizing enterocolitis, inflammation, AOC3 protein, Intestines Cell adhesion molecules

## Abstract

**Background:**

Necrotizing enterocolitis (NEC) is an inflammatory bowel disease in preterm neonates with high morbidity and mortality. The only treatment available is supportive with broad-spectrum antibiotics and gastrointestinal rest. Better understanding of the pathogenesis is crucial for the development of new therapies. Vascular adhesion protein-1 (VAP-1), expressed in human blood vessels and lymphatic, plays a crucial role in the pathogenesis of inflammatory diseases in adults. The aim of the study was to investigate the VAP-1 expression in the intestines of infants affected by NEC.

**Methods:**

Intestinal tissues from 42 preterm infants with NEC were examined with immunohistochemical staining using antibodies against VAP-1 and semi-automated digital image analysis was performed to determine tissue protein expression of VAP-1 in blood vessels located in the submucosa. Intestinal tissue from 26 neonates that underwent laparotomy and ileostomy due to other intestinal surgical conditions served as controls. Clinical data and protein expression were compared between the NEC-group and Controls.

**Results:**

Mean gestational age was lower in NEC infants compared to controls, 26.6 ± 3.0 gestational weeks versus 36.5 ± 4.0 (*p* < 0.001) but without any significant difference in median postnatal age at surgery; for NEC 8 (5–27) days and for controls 3 (1–36) days (*p* = 0.6). Low VAP-1 correlated with increased risk for developing NEC in the logistic regression (*p* < 0.001). Multiple linear regression showed that both gestational age and NEC were independent predictors of VAP-1 expression.

**Conclusion:**

VAP-1 may play a role in the pathogenesis of NEC. Diminished expression of VAP-1 independent of maturation could indicate arrested vascular development in infants suffering from NEC. Further studies are needed to elucidate the role of VAP-1 in NEC.

**Supplementary Information:**

The online version contains supplementary material available at 10.1186/s12887-022-03681-9.

## Introduction

Necrotizing enterocolitis (NEC) is a life-threatening disease that predominantly affects preterm infants [1, 2]. The risk of NEC is inversely related to birth weight and gestational age (GA) [1]. Advances in neonatal intensive care have improved survival rates among extremely preterm infants which subsequently has increased the population at risk of developing NEC [1–3].

Despite decades of research, the only available treatment of NEC is bowel rest and broad-spectrum antibiotics. Nevertheless, 50% of the infants need emergency surgery which is associated with high mortality rates (30–50%), intestinal failure and neurodevelopmental impairment [1, 2, 4–8].

NEC was first described in 1965 [9]. Ever since then several attempts have been made to understand the etiology and pathogenesis of the disease. Current evidence suggests a multifactorial cause of NEC, with both prenatal and postnatal factors [10, 11]. Prematurity is the main risk factor, presumably due to the immaturity of gastrointestinal motility, the intestinal barrier function and immune defences [1, 11]. NEC is characterized by inflammation of the intestines and has been associated with imbalanced gut microbiota development, primarily Gamma-proteobacteria [10, 12]. It is also suggested that the intestinal inflammation is amplified by abnormal microcirculation of the preterm infant [13].

An association exists between oxidative stress and inflammation, and oxidative stress has been shown to play a role in the pathogenesis of NEC [14–16]. Newborns and especially preterm infants are more exposed to oxidative stress than adults and children [14].

Vascular adhesion protein-1 (VAP-1) is a member of the copper-containing amine oxidase/semicarbazide-sensitive amine oxidase enzyme family and is expressed as a transmembrane glycoprotein in the vascular wall [17]. VAP-1 is produced in endothelial cells, smooth muscle cells and adipocytes [18] and is notably expressed in human blood vessels under physiological conditions [19, 20]. During inflammation, endothelial VAP-1 is upregulated and its enzymatic activity is indispensable for leukocyte extravasation through endothelium to the tissue [21]. The enzymatic actions of VAP-1 on leukocytes is mediated by the production of hydrogen peroxide [18, 21] that is produced during oxidative stress [14–16]. Altered expression of VAP-1 is involved in the pathogenesis of several inflammatory diseases in adult humans [18, 22, 23].

The aim of the study was to investigate the VAP-1 expression in the intestines of preterm infants affected by NEC.

## Material and methods

### Study population

Preterm infants treated at the neonatal intensive care unit of the Uppsala University Children’s Hospital, Uppsala, Sweden between September 2003 and April 2019 and who underwent laparotomy due to NEC or other diagnosis (controls) were recruited for the study. The study protocol was approved by the Regional Ethical Review Board (Dnr 2019–00437) and written informed consent was obtained from the parents.

NEC was diagnosed by radiological and clinical features and staged according to the criteria of Bell et al. [24]. Necrotic bowel was resected at surgery and NEC diagnosis was confirmed during surgery and by histopathological evaluation. Only samples that represented macroscopically vital tissue from the ends of the resected intestine were selected for further histopathological evaluation.

Infants with intestinal atresia, dysmotility due to intestinal immaturity, aganglionisis, pseudo-obstruction or volvulus served as controls. They underwent laparotomy between 0 and 78 days of life. Samples from the controls were taken from the site of the stoma. Patient data was extracted from the medical records.

### Intestinal tissue samples and immunohistochemistry

All samples were sectioned and stained on the same occasion for comparable analysis. Paraffin-embedded sections were placed in a PT Link Pre-treatment Module (Agilent) 97 °C for 20 minutes for deparaffinization, followed by incubation with Target Retrieval Solution, Citrate pH 6 (S236984–2, Agilent) for 30 minutes. Immunohistochemistry was performed in an Autostainer Link 48 (Agilent) using an EnVision FLEX visualization system (Agilent) and counterstained with hematoxylin-eosin. Tissue sections were incubated for 30 minutes at room temperature with primary antibody, a polyclonal rabbit anti-human VAP-1 antibody (1:100, PA5–81910 Thermo Fischer Scientific). Negative control sections were prepared by performing immunostaining procedures without adding primary antibodies. Stained sections were scanned by a digital slide scanner (NanoZoomer S60, Hamamatsu) using the same exposure times. Digitalized sections were examined by NDP.view2 (Hamamatsu), a whole slide viewing software. The same magnification (10 x objective) was used for all the images. Three representative areas per section/patient were exported into three images (size, 23 MP, 6400 × 3616 pixels, type: RGB, format: TIFF). RGB image allowed the range of 255 intensity levels in the three color channels (red, green, blue), no saturated pixels could be observed.

### Software and image analysis

Blood vessels were manually selected in the submucosa by identification of typical histological features (Supplemental Fig. 1). Images were manually selected, a total of 204 images were sorted into stacks and saved in TIFF format. From the image regions of interest (ROI), areas containing blood vessels, were identified and selected manually (*n* = 280). ImageJ [25] was used for semiautomatic image analysis. Higher VAP-1 expression corresponded to higher pixel intensity. Before analysis, the same threshold window was set on all images in order to exclude unspecific low pixel values.

Both the immunohistochemistry and the image analysis were performed blinded.

### VAP-1 expression

The analysis of VAP-1 expression was performed using the Analyze Particles tool in ImageJ. Five variables were chosen to describe the characteristics of the digital images.

- VAP-1 area %: number of pixels in the threshold covered area divided by the total amount of pixels in the image area;
- VAP-1 mean pixel intensity: mean value of pixel intensity level within the threshold area;
- VAP-1 median pixel intensity: median intensity value within the threshold area;
- VAP-1 mode pixel intensity: most frequently occurring pixel intensity value within the threshold area and;
- VAP-1 max pixel intensity: maximal intensity value within the threshold area.

All the five parameters were estimated for each individual image. The mean values of the corresponding ROI images were calculated in Microsoft Excel. VAP-1 area % was chosen for further analysis as it found to be the most accurate indicator for visual VAP-1 expression (Supplemental Fig. 2). Furthermore, this was the most complex parameter describing VAP-1 expression: it corresponds indirectly to blood vessel density and strongly correlates with intensity levels of VAP-1 (VAP-1 mean pixel intensity) in blood vessels (Supplemental Fig. 3).

### Cluster analysis

Clinical data (GA, birth weight, postmenstrual age (PMA) at surgery and postnatal age (PNA) at surgery) were analyzed and automatically sorted by two-dimensional hierarchical clustering (Cluster 3.0 freeware [26]) as previously described [27, 28]. The patients were sorted into groups, and the line between the groups were identified both by visual evaluation and the help of the tree diagrams. All clinical parameters were used and weighed equally in the clustering algorithm. Results were visualized using Java TreeView [29, 30].

### Statistical analysis

Statistical analysis was performed in R-studio version 1.4.1717 and Microsoft Excel 2016 version (16.0.13901.20400). *P*-values were considered statistically significant when < 0.05. All tests of significance were two-tailed. VAP-1 expression was compared using Student’s *t*-test. Linear regression analysis was performed using Pearson’s correlation. Multiple linear regression analysis was performed to determine whether GA and NEC could predict VAP-1 expression. One-way ANOVA with Tukey’s HSD post-hoc test was performed to compare VAP-1 expression between groups.

## Results

### Clinical characteristics of the study population

**Fig. 1:**
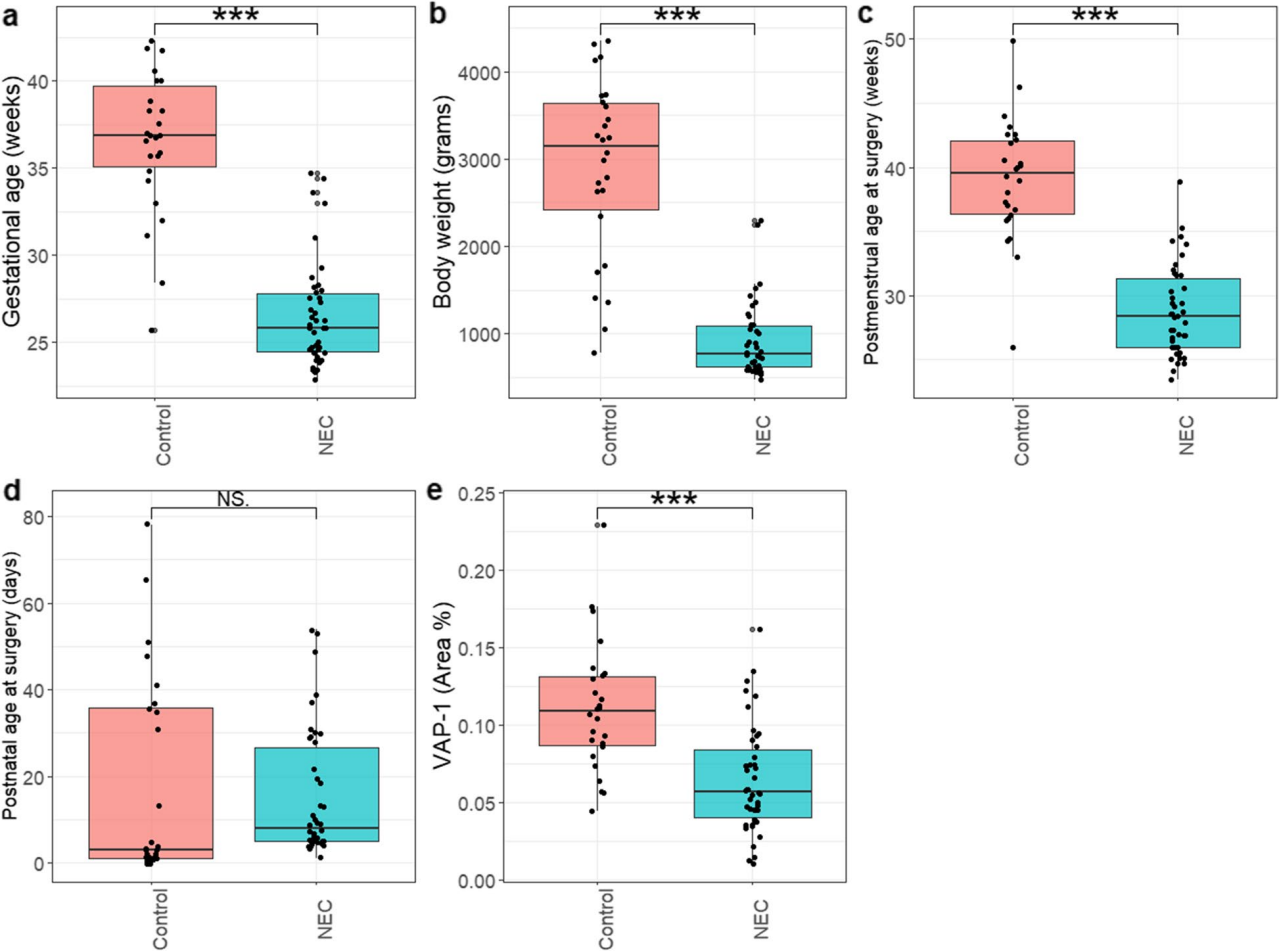
There was a significant difference in (**a**) gestational age (**b**) body weight and (**c**) postmenstrual age between the NEC group and controls. **d** There was no significant difference in postnatal age at surgery. **e** VAP-1 expression was significantly lower in the NEC group. NS signifies *p* > 0.05, * signifies *p* < 0.05, ** signifies *p* < 0.01 and *** signifies *p* < 0.001

A total of 68 infants were included in the study. The NEC group consisted of 42 infants (26 males and 16 females) and the control group of 26 infants (14 males and 12 females). Mean GA for all infants was 30.4 ± 5.9 weeks and the mean body weight was 1678 ± 1195 g. The mean GA was lower in the NEC group compared to controls 26.6 ± 3.0 gestational weeks versus 36.5 ± 4.0 (*p* < 0.001) (Fig. 1A). The mean body weight was lower in the NEC group, 918 ± 424 g compared to the controls 2906 ± 1026 g (*p* < 0.001) (Fig. 1B). PMA at surgery was also found to be lower in the NEC group, 28.8 ± 3.5 gestational weeks compared to 39.1 ± 4.7 gestational weeks in the control infants (*p* < 0.001) (Fig. 1C). There was no significant difference in PNA at surgery; median PNA for NEC was 8 (5–26) days, and for controls 3 (1–36) days (*p* = 0.6) (Fig. 1D).

### VAP-1 expression

**Fig. 2:**
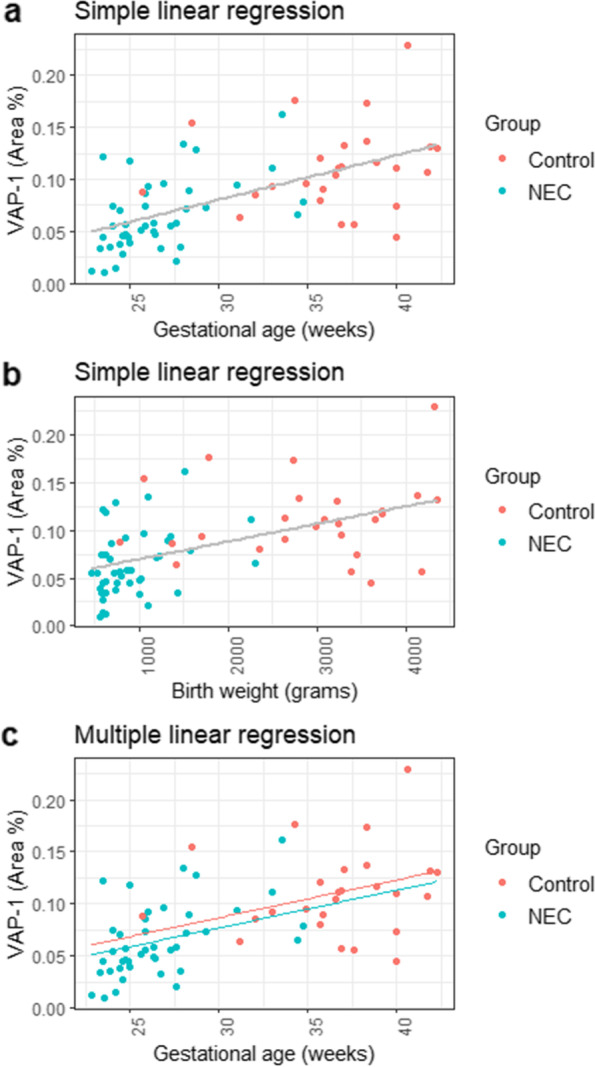
Linear regression analysis showed that both (**a**) gestational age and (**b**) body weight could predict VAP-1 Area %. **c** Both gestational age and NEC were independent predictors of VAP-1 Area %

The staining showed high specificity to label blood vessels in the submucosa. VAP-1 area % correlated with blood vessel density in the tissue. Mean, median, mode, max pixel intensities described staining characteristics within the ROI and the threshold area, independently to the absolute number of blood vessels. These four variables allowed comparing patients with diverse blood vessel densities. VAP-1 area % was found to be significantly lower in the NEC group (*n* = 26) compared to controls (*n* = 16) (*p* < 0.001) (Fig. 1E). Simple linear regression indicated that both GA and body weight could predict VAP-1 expression as shown in Fig. 2 A-B. Categorical linear regression indicated a significant correlation between the presence of NEC and VAP-1 expression (*R* = 0.52, F (1, 66) = 23.88, *p* < 0.05).

Multiple linear regression was performed to evaluate whether GA and the presence of NEC could predict VAP-1 expression. GA and NEC were independent predictors of VAP-1 expression (Fig. 2C).

In total 12 infants out of 68 died. There was no correlation found between VAP-1 expression and all type mortality (*p* = 0.17). 11 out of 42 NEC infants died and there were no significant difference in VAP-1 expression between the two groups (*p* = 0.99) (Supplemental Fig. 4). NEC infants with high VAP-1 expression (VAP-1 area % > 0.056) had similar mortality to NEC infants with low VAP-1 expression (VAP-1 area % < 0,056) (*p* = 0.73).

### Identification of clinical groups with cluster analysis

**Fig. 3:**
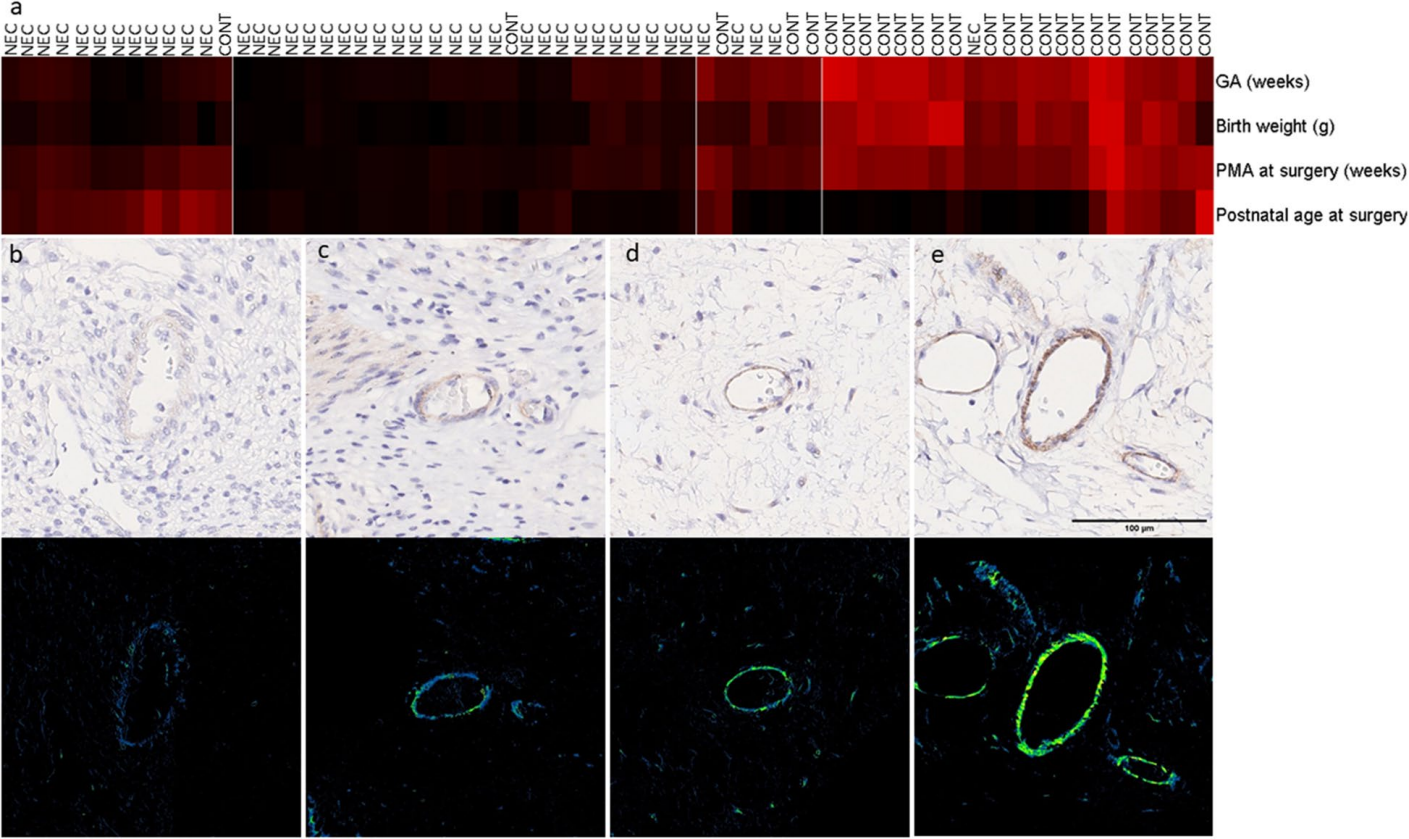
Two dimensional hierarchical clustering generated four clinical groups with visually different microscopic patterns. **a** Higher intensity of red colour indicates a higher expression of a given clinical parameter. **b** Microscopic image samples from group 1, (**c**) group 2, (**d**) group 3 and (**e**) group 4. Higher VAP-1 expression is indicated by increased green-blue pixel intensity. GA (gestational age), PMA (postmenstrual age), PNA (postnatal age)

Investigating patients in multiparametric subgroups allowed us to uncover differences in VAP-1 expression due to NEC status and maturation level. Two-dimensional hierarchical clustering of clinical parameters (GA, body weight, PMA at surgery and PNA at surgery) was performed and identified four unique clinical groups (Fig. 3A) and demonstrated how VAP-1 varies based on maturity within the NEC- and control group. Each group displayed different expression levels of VAP-1 in blood vessels (representative regions of the groups are shown in Fig. 3B-E).

**Fig. 4:**
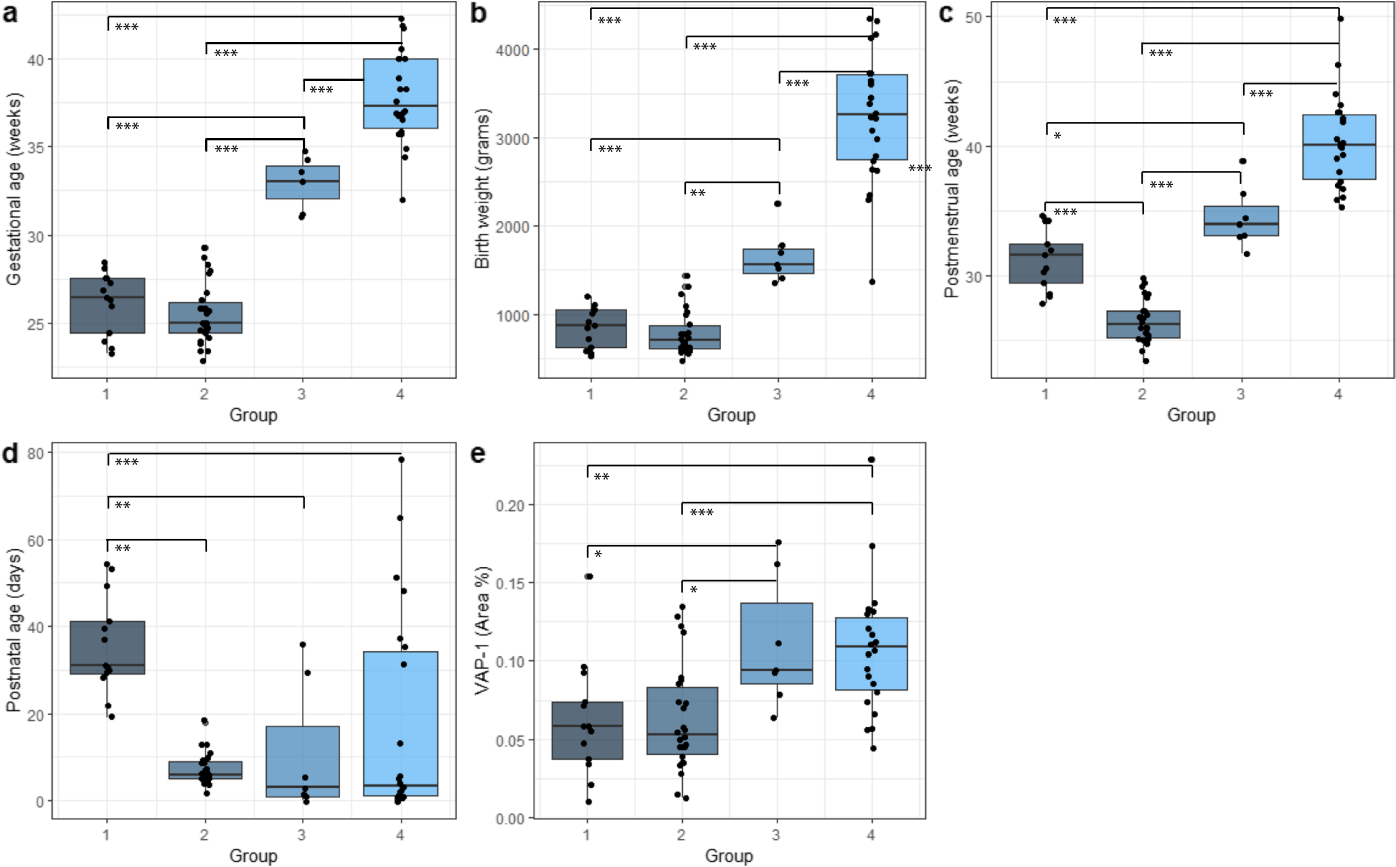
The four groups display unique clinical patterns, and the VAP-1 expression varies between clinical groups. One-way ANOVA revealed that there was a statistically significant difference in (**a**) gestational age, (**b**) body weight, (**c**) postmenstrual age at surgery and (**d**) postnatal age at surgery between at least two groups. **e** One-way ANOVA revealed statistically significant difference between at least two groups for VAP-1 Area %. * signifies *p* < 0.05, ** signifies *p* < 0.01 and *** signifies *p* < 0.001

NEC- and control infants were automatically sorted into the following groups using the cluster analysis method (Figs. 3A, 4 and Table 1): Group 1 (*n* = 13; NEC = 12, controls = 1), preterm NEC infants at 27–34 weeks PMA at surgery; Group 2 (*n* = 26; NEC = 25, controls = 1), preterm NEC infants at 23–29 weeks PMA at surgery; Group 3 (*n* = 7; NEC = 4, controls = 3), preterm infants at 31–38 weeks PMA at surgery; Group 4 (*n* = 22; NEC = 1, controls = 21), term control infants. Significant differences in GA, birth weight, PMA and PNA at surgery were found (Fig. 4A-D) (*p* < 0.05.). VAP-1 expression was also found to be significantly different between groups (Fig. 4E). NEC infants in group 1 had higher PNA and PMA at surgery when compared with group 2. On average, infants in group 1 were operated 4.7 weeks later than in group 2 (Table 1). However, there were no significant differences in VAP-1 expression between the two groups. Tukey HSD post hoc test showed significant differences between groups as shown in Table 2.

**Table 1:** Characteristics of subgroups

	Gestational age (weeks)	Birth weight (grams)	Postmenstrual age at surgery (weeks)	Postnatal age at surgery (days)	VAP-1 (Area %)
Group 1	26.1 ± 1.8	850 ± 228	31.2 ± 2.3	35.5 ± 11.2	0.062 ± 0.037
Group 2	25.5 ± 1.7	782 ± 254	26.5 ± 1.7	7.04 ± 3.6	0.063 ± 0.034
Group 3	33.0 ± 1.4	1655 ± 302	34.5 ± 2.4	10.7 ± 15.1	0.11 ± 0.42
Group 4	37.8 ± 2.7	3233 ± 737	40.4 ± 3.6	17.7 ± 24.0	0.11 ± 0.014

Subgroups were identified by two dimensional hierarchical cluster analysis. Results are reported as means and standard deviations. Results are reported as means ± standard deviation

**Table 2:** Additional multiple comparisons of all groups to test for differences detected by the cluster analysis

	Gestational age	Birth weight	Postmenstrual age at surgery	Postnatal age at surgery	VAP-1 (Area %)
Group 1 vs 2	0.81	0.97	***	***	0.99
Group 1 vs 3	***	**	*	**	*
Group 1 vs 4	***	***	***	**	**
Group 2 vs 3	***	**	***	0.94	*
Group 2 vs 4	***	***	***	0.09	***
Group 3 vs 4	***	***	***	0.72	0.99

Tukey HSD was used to determine differences between groups. *p*-values > 0.05 indicate that two groups are statistically different from each other in the specific parameter. * signifies *p* < 0.05, ** signifies *p* < 0.01 and *** signifies *p* < 0.001

## Discussion

During recent years there has been an increase in NEC as cause of death in preterm infants with mortality rates reaching 30–50% in advanced cases [31–34]. Besides substantial mortality and morbidity in survivors of NEC [1, 2, 4–8], the disease is associated with a longer hospital stay and high medical costs [35, 36]. Each case of medical or surgical NEC costs 70,000/200000 USD [35] and prolongs hospital stay (+ 60–80 days) [36]. The treatment of NEC is still only supportive and efficient therapies are needed. Hence, better understanding of the pathogenesis of NEC is crucial for the development of new treatment methods.

To our knowledge, this is the first study of VAP-1 expression in the intestines of preterm infants. Previous studies have shown elevated levels of circulating soluble VAP-1 in adult patients with inflammatory diseases [18, 23]. In 2001 Salmi et al [37] demonstrated that gut-derived leucocytes from patients with ulcerative colitis and Crohn’s disease were found to bind well to venules in synovial membrane [37]. Blocking of VAP-1 significantly inhibited the binding of all leukocyte subsets to joint vessels, suggesting an important role of VAP-1 in intestinal inflammation and the development of reactive arthritis in inflammatory bowel disease [37]. In the present study we found that preterm infants affected by NEC had a lower expression of VAP-1 in the blood vessels of the intestines compared to controls.

It has been concluded that the most significant clinical risk factor for NEC is low GA [1]. Our results show that VAP-1 expression could be predicted by GA. Similarly, the presence of NEC was correlated with lower levels of VAP-1 expression. One could argue that the correlation between VAP-1 and NEC is due to the covariation between NEC and GA However, we found no difference in VAP-1 expression between group 1 and group 2. Both of these groups consisted of mainly NEC infants but with different PNA and PMA at surgery. Summarized, VAP-1 expression did not increase during the postnatal maturation of the bowel in patients with NEC (group 1). We speculate that it could be explained by arrested vascular development in infants suffering from NEC. Moreover, multiple regression analysis showed that the addition of NEC as a categorical parameter to the regression model generated a stronger correlation coefficient. Strengthening the hypothesis that NEC in itself is a significant predictor of VAP-1 expression.

As previously described [10, 12] infants with NEC have an altered intestinal microbiota. Lymphocyte migration between blood and tissue is crucial in mounting proper immune responses. Recirculation and mobilization of lymphocytes is required for dispersal of effector lymphocytes into peripheral organs and for contacts between antigen-presenting cells and responding lymphocytes in secondary lymphoid organs. The generation of microbial immune response is the most vital function of this system [38]. One of the main functions of VAP-1 is as an adhesion molecule, assisting in the migration of leukocytes from the bloodstream into the tissue [18, 39]. Recently, VAP-1 deficient mice have been shown to display paucity in Peyer’s patches of the gut and have an impaired response to infections with *Staphylococcus aureus* and Coxackie B4 (37). The mice also displayed impaired immune responses after oral vaccination. In summary, the VAP-1 deficient mice displayed several traits that would suggest that the lack of VAP-1 caused a weakened immune response [38]. The absence of VAP-1 may be associated with a mild defect in lymphocyte recirculation under physiologic conditions or an impaired host response upon inflammatory challenge [20, 21]. Accordingly, low VAP-1 expression in the intestines of preterm infants may result in an impaired immune response. As a result of the weakened immune system, the infants could develop an imbalanced gut microbiota and intestinal inflammation as found in NEC.

The strengths of this study were the inclusion of a large sample size with intestinal tissue from infants with NEC and control infants that underwent laparotomy due to other intestinal surgical conditions. Moreover, both immunohistochemistry and image analysis was performed blinded increasing the internal validity. A limitation of our study was that the control group of infants were not completely healthy and had a higher GA. However, it was not possible to find a better control group since it was not ethically defensible to take tissue samples from completely healthy infants. There was a higher number of males in the NEC group. This could lower the generalizability of our results. However, previous studies have not been able to conclude that gender is a risk factor for developing NEC [40].

## Conclusion

These results suggest that VAP-1 may play a role in the pathogenesis of NEC as we found a correlation between low VAP-1 expression in the blood vessels of the intestines in infants with NEC. Both NEC disease and GA could independently predict VAP-1 expression. VAP-1 expression did not increase during the postnatal maturation of the bowel in patients with NEC. Diminished expression of VAP-1 could indicate arrested vascular development in infants suffering from NEC. Further studies are needed to elucidate the role of VAP-1 in NEC.

## Supplementary Information

**Additional file 1: Supplementary Fig. 1.** Microscopic image of a representative tissue sample with original magnification.

**Additional file 2: Supplementary Fig. 2.** Significant difference in VAP-1 expression. There were significant differences in **a)** VAP-1 area % (NEC = 0,065 ± 0,035; controls = 0,11 ± 0,042, t(1,66) = 4.89, *p* < 0.001), **b)** VAP-1 mean (NEC = 80,74 ± 3,81; controls = 86,29 ± 6,06, t(1,66) = 4.65, *p* < 0.001), **c)** VAP-1 median (NEC = 76,44 ± 3,39; controls = 81,92 ± 6,09, t(1,66) = 4.77, *p* < 0.001), **d)** VAP-1 mode (NEC = 64,79 ± 1,07; controls = 62,33 ± 4,09, t(1,66) = 3.72, *p* < 0.001) and **e)** VAP-1 max (NEC = 150,99 ± 15,3; controls = 162,45 ± 12,3, t(1,66) = 3.23, *p* < 0.01). * signifies *p* < 0.05, ** signifies *p* < 0.01 and *** signifies *p* < 0.001.

**Additional file 3: Supplementary Fig. 3.** Linear regression indicated a strong correlation between mean VAP-1 expression and VAP-1 area % for a) all included infants (*R*^2^ = 0.61, F(1, 66) = 101.4, *p* < 0.001, *n* = 68), b) NEC infants (*R*^2^ = 0.52, F(1, 40) = 43.69, *p* < 0.001, *n* = 42) and for c) control infants (*R*^2^ = 0.49, F(1, 24) = 22.88, *p* < 0.001, *n* = 26).

**Additional file 4: Supplementary Fig. 4.** Difference in VAP-1 expression between alive and deceased NEC infants. No significant difference in VAP-1 Area % was found (Alive = 0.065 ± 0.034; Deceased = 0.065 ± 0.038, t(1,40) = − 0.0061, *p* = 0.99). NS signifies *p* > 0.05.

## Data Availability

The datasets used and/or analysed during the current study are available from the corresponding author on reasonable request.

## Abbreviations

VAP-1: Vascular adhesion protein – 1
NEC: Necrotizing enterocolitis
GA: Gestational age
PNA: Postnatal age
PMA: Postmenstrual age
ROI: Region of interest
