# Supplementary figures and images for: Vascular adhesion protein-1 expression is reduced in the intestines of infants with necrotizing enterocolitis: an observational research study
Source: BMC Pediatr. 2022 Nov 5;22:640. doi: 10.1186/s12887-022-03681-9 (PMC9636710; doi:10.1186/s12887-022-03681-9)

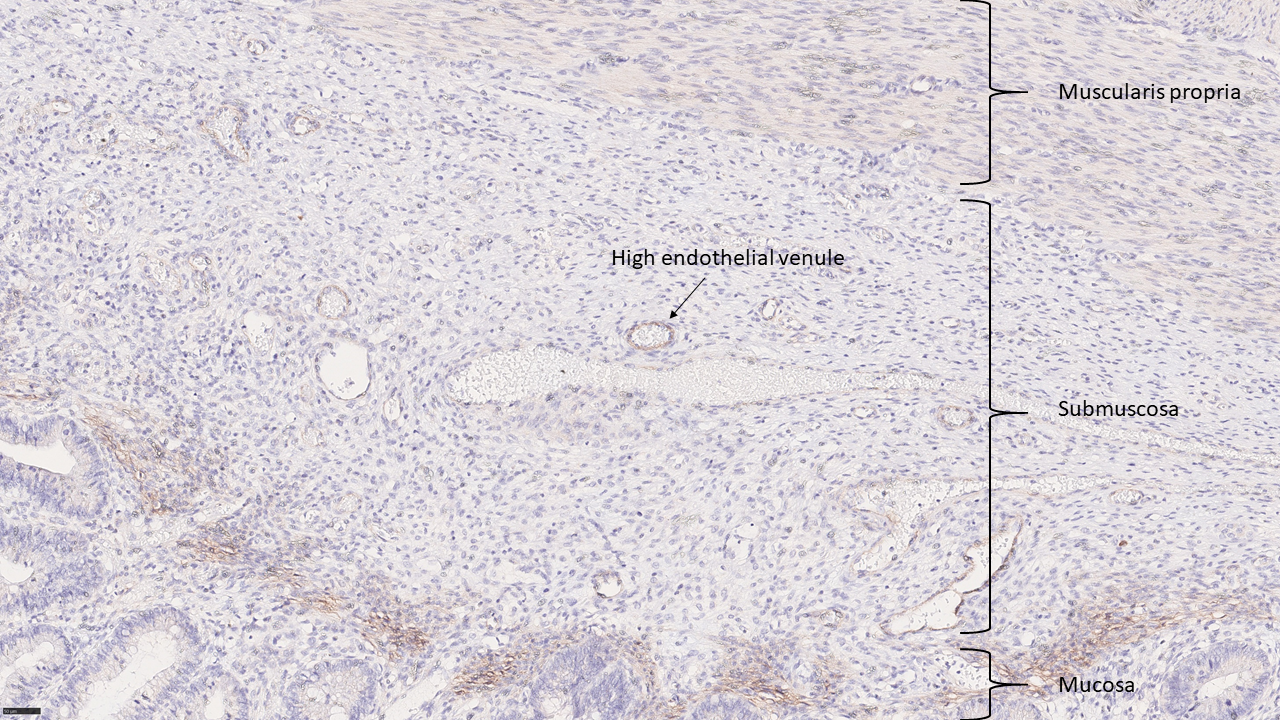

Supplement: Supplementary file 1 — Additional file 1: Supplementary Fig. 1. Microscopic image of a representative tissue sample with original magnification. [file 12887_2022_3681_MOESM1_ESM.png]

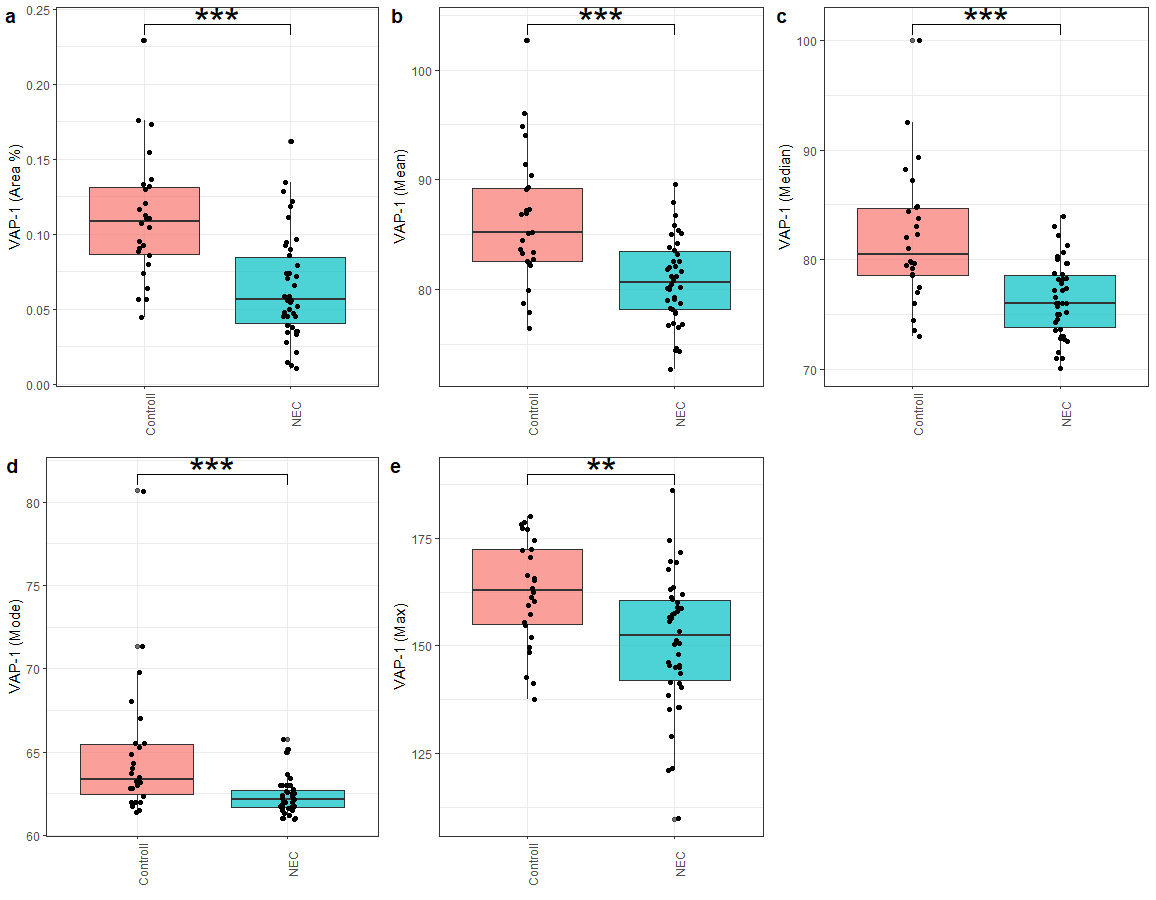

Supplement: Supplementary file 2 — Additional file 2: Supplementary Fig. 2. Significant difference in VAP-1 expression. There were significant differences in a) VAP-1 area % (NEC = 0,065 ± 0,035; controls = 0,11 ± 0,042, t(1,66) = 4.89, p < 0.001), b) VAP-1 mean (NEC = 80,74 ± 3,81; controls = 86,29 ± 6,06, t(1,66) = 4.65, p < 0.001), c) VAP-1 median (NEC = 76,44 ± 3,39; controls = 81,92 ± 6,09, t(1,66) = 4.77, p < 0.001), d) VAP-1 mode (NEC = 64,79 ± 1,07; controls = 62,33 ± 4,09, t(1,66) = 3.72, p < 0.001) and e) VAP-1 max (NEC = 150,99 ± 15,3; controls = 162,45 ± 12,3, t(1,66) = 3.23, p < 0.01). * signifies p < 0.05, ** signifies p < 0.01 and *** signifies p < 0.001. [file 12887_2022_3681_MOESM2_ESM.png]

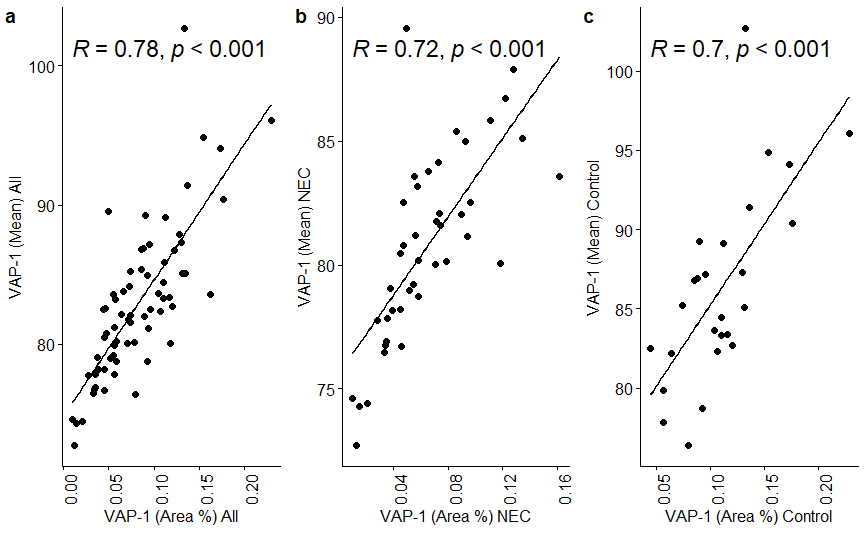

Supplement: Supplementary file 3 — Additional file 3: Supplementary Fig. 3. Linear regression indicated a strong correlation between mean VAP-1 expression and VAP-1 area % for a) all included infants (R2 = 0.61, F(1, 66) = 101.4, p < 0.001, n = 68), b) NEC infants (R2 = 0.52, F(1, 40) = 43.69, p < 0.001, n = 42) and for c) control infants (R2 = 0.49, F(1, 24) = 22.88, p < 0.001, n = 26). [file 12887_2022_3681_MOESM3_ESM.png]

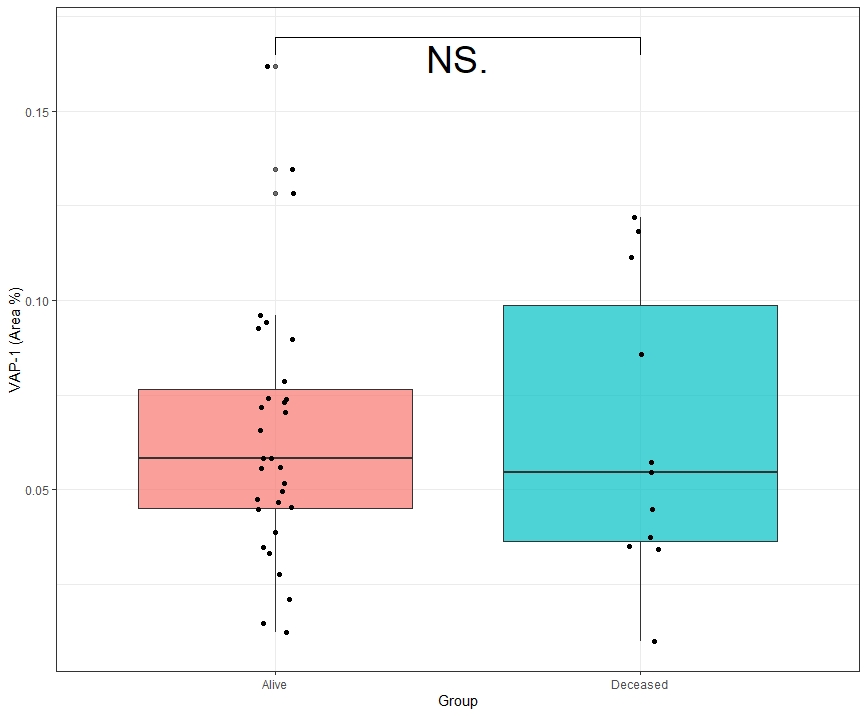

Supplement: Supplementary file 4 — Additional file 4: Supplementary Fig. 4. Difference in VAP-1 expression between alive and deceased NEC infants. No significant difference in VAP-1 Area % was found (Alive = 0.065 ± 0.034; Deceased = 0.065 ± 0.038, t(1,40) = − 0.0061, p = 0.99). NS signifies p > 0.05. [file 12887_2022_3681_MOESM4_ESM.jpeg]
